# Changes of temporomandibular joint position after surgery first orthognathic treatment concept

**DOI:** 10.1038/s41598-019-38786-2

**Published:** 2019-02-18

**Authors:** Daniel Holzinger, Katrin Willinger, Gabriele Millesi, Kurt Schicho, Elisabeth Breuss, Florian Wagner, Rudolf Seemann

**Affiliations:** 1 Department of Oral and Maxillofacial surgery, Medical University of Vienna, Waehringer Guertel 18-20, 1090 Vienna, Austria; 2 University Clinic of Dentistry, Medical University of Vienna, Sensengasse 2a, 1090 Vienna, Austria

## Abstract

Orthognathic surgery treatment (OGS) after orthodontic treatment of dentofacial deformities is a widely performed procedure, often accompanied by a bilateral sagittal split osteotomy (BSSO). Positioning of the condyle during this procedure is a crucial step for achieving optimal functional and anatomical results. Intraoperatively poorly positioned condyles can have a negative effect on the postoperative result and the patient’s well-being. Changes of the condylar position during OGS Procedures and its effects on the temporomandibular joint in orthognathic surgical interventions (OGS) are subject of scientific discussions. However, up to date, no study has investigated the role of condyle position in the surgery first treatment concept. The aim of this study was to investigate the influence of OGS on the three-dimensional position of the condyle in the joint in a surgery first treatment concept without positioning device and to record the change in position quantitatively and qualitatively. Analysis of our data indicated that OGS in surgery first treatment concept has no significant effect on the position of the condyle and the anatomy of the temporomandibular joint.

## Introduction

In patients with dentofacial deformities (DFD) and temporomandibular joint disorders (TMD), orthognathic surgery (OGS) is performed to improve masticatory function and aesthetics.

It is known, that orthognathic surgery affects the temporomandibular joint (TMJ); how DFD and TMD effect each other is still poorly understood and in the focus of research^1,2^.

A variety of studies have reported that temporomandibular joint (TMJ) or temporomandibular disk displacement causes skeletal Class II deformities as well as hyper-divergent facial deformities. These deformities are likely to progress with age^3^, and can even cause mandibular midline asymmetry^1^, if left untreated^2,4,5^.

Thus, the role of orthognathic surgery in DFD and TMD needs to be further investigated^6–8^ OGS procedures aim to resolve these issues by means of mono- or bimaxillary osteotomy of the facial skeleton accompanied by orthodontic treatment.

Only very few studies elucidate the long-term impact of orthognathic surgery on the temporomandibular joints’ structure and function^9,10^. Some authors postulate that mispositioning of the condyle after bilateral sagittal split osteotomy (BSSO) of the mandible leads to morphologic changes of the condyle, temporomandibular dysfunction or skeletal relapse^11–14^. A change in the position of the condyle may occur during surgery for any number of reasons. The recumbent position of the patient, paralysis of masticatory muscles due to anesthetization, joint edema, malalignment of bone ligaments, methods of positioning the condyle, and fixation methods can all lead to deviations in position of the condyle. It has been shown that our ability to reproduce condylar position even when no surgery is performed can be altered by patient position and relaxation of the masticatory muscles. Hence, numerous devices have been designed to maintain the preoperative position of the condyle during orthognathic surgery^15,16^.

Despite the rich literature and numerous disputes on the effects of orthognathic surgery especially regarding DFD/TMD, there are only a few reports that focus on the changes and symptoms of the condyle positions after orthognathic surgery. It is still unclear how orthognathic surgical procedures change the condylar position, and whether these changes vary significantly from the preoperative TMJ position^14^. Minor positional adjustments of the condyle can be characterized and quantified by three-dimensional imaging, including conventional fan-beam computed tomography (CT) or cone-beam computed tomography (CBCT)^17^.

This study aims to clarify if there are significant changes in condyle position after free hand repositioning of the condyle during OGS, since these changes may affect the function of the TMJ and impair masticatory function of the patients after surgery. We postulate that free hand repositioning does not alter the condyle position after surgery.

## Materials and Methods

This prospective study was conducted after approval of the Ethics Committee of the Medical University of Vienna, Austria (1449/2013) and is in compliance with the Helsinki Declaration. Written, informed consent was obtained from all patients that were included in our study. The study was carried out on CT scans of patients >18 years of age, suffering from front open bite. The patients were operated between June 2013 and August 2014 at the Department of Oral and Maxillofacial Surgery of the Medical University of Vienna. All patients had undergone orthognathic surgery before orthodontic treatment, and all operations were conducted by an experienced surgeon. Maxillary treatment was performed doing a Le Fort I osteotomy, using four L-shaped mini-plates with four screws each for fixation. Mandibular treatment was done by the standard bilateral sagittal split osteotomy (BSSO) technique according to Hunsuck and Epker^18,19^. All condyle bearing segments were positioned free-hand without positioning devices, using three bicortical setscrews with lengths from 12 mm–16 mm for rigid fixation.

### Data Collection

A CT scan of the skull (Philips Brilliance 64, Amsterdam, Holland; technical data see Table 1) was performed before surgery according to a standardized protocol with the patient in natural head position and gently biting in centric relation, resting his lips in a relaxed position. All data were saved in the Vienna General Hospital (Allgemeines Krankenhaus Wien, AKH) Picture Archiving Communication System (PACS), and later saved on a CD Rom for data analysis.

**Table 1:** Technical data of CT Philips Brilliance® 64.

	Philips Brilliance® 64
Tube voltage (kV)	80, 120, 140 kV
Tube current (mA)	20–500 mA
Detector elements	43.008
Gantry opening	700 mm
Gantry slope	−30/+30 increment 0.5
Scan time	Up to 100 sec
Scan length	1750 mm
Slice thickness	0.55–7.5 mm
Rotation time	0.4, 0.5, 0.75, 1, 1.5, 2 sec for 360° scans 0.28, 0.33 sec for 240° scans
pitch	0.13–1.5
collimation	64 × 0.625 mm, 40 × 0.625 mm, 32 × 1.25 mm, 16 × 2.5 mm, 2 × 0.5 mm
Field of view (FOV)	250 or 500
Image/sec	20

Each operation was performed according to a standardized protocol by two experienced surgeons using a traditionally fabricated surgical splint for guidance.

Six months after surgery, another CT scan was performed according to the protocol described in Table 1. The condyle position was evaluated bilaterally and compared with the postoperative CT with the program Slicer 3D (V.4.4.0 http://www.slicer.org/)^20^, reconstructing three-dimensional datasets for quantitative morphometric analysis.

**Figure 1:**
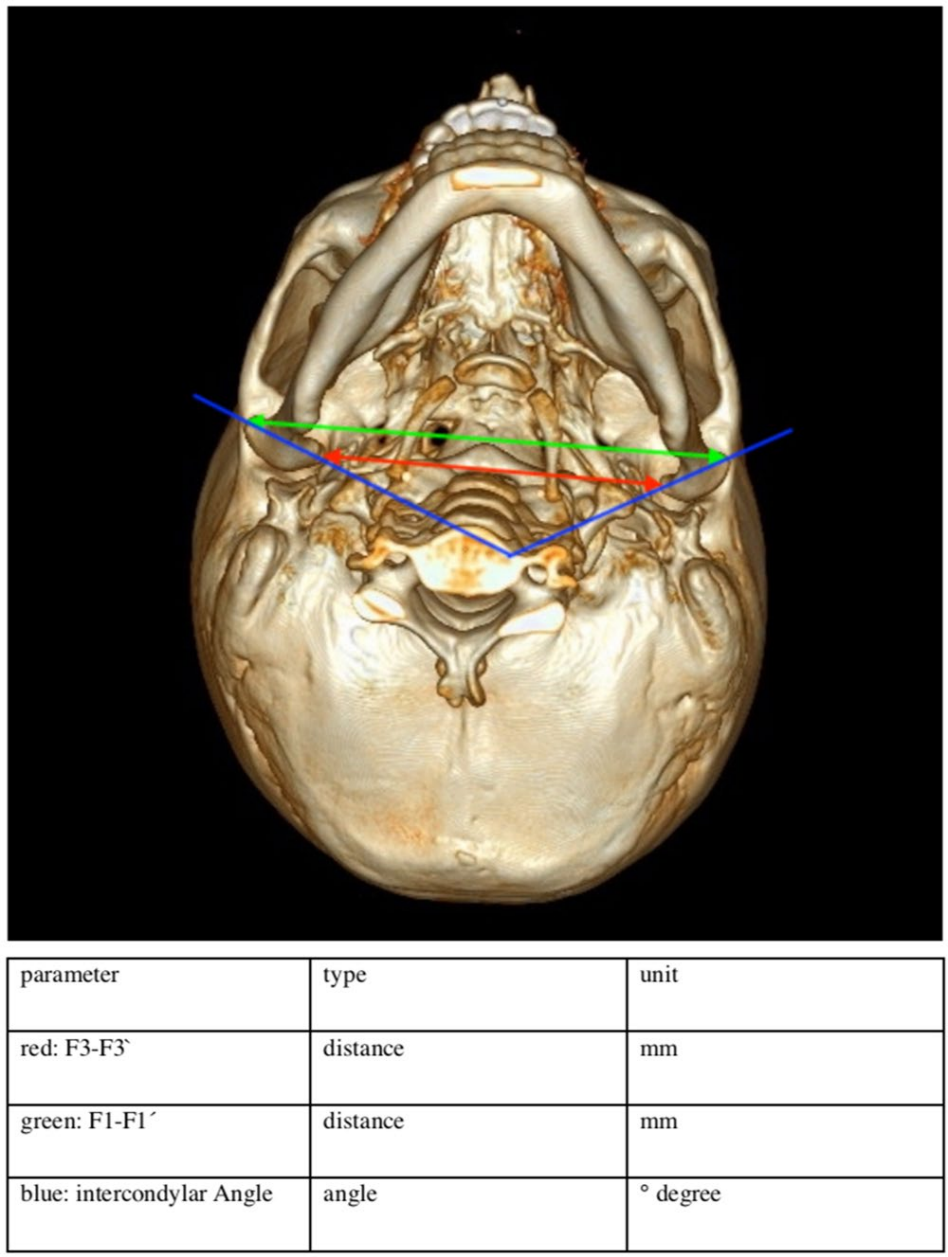
Variables assessed for quantitative morphological analysis.

Only bone kernel data visible to the human eye (Table 2) were used for reconstructing a raw data set of the mandible. Data were thresholded, thus Hounsfield Unit scale (HU) below 60 are projected in white color, and all parameters above 80 HU are projected in black color. Alterations of the TMJ were quantified by determining preoperative to postoperative differences between previously defined adjacent anatomical landmarks: F1(right), F1′(left)- most lateral point of the condyle, F2(right), F2′(left) - most anterior point of the condyle, F3 (right), F3′(left) - most medial point of the condyle, F4(right), F4′(left) - most posterior point of the Condyle. Then distance was measured between the most medial point of the condyles (F3-F3′) and between the most lateral point of the condyles (F1-F1′). In addition, the distance of both condylar longitudinal axes was calculated and the cutting angle between the axes (Intercondylar Angle) was calculated and measured. All measurements are in millimeters (mm), Angles are measured in degrees (see Fig. 1).

**Table 2:** Exam parameters used for quantitative analysis.

Tube voltage (kV)	120–140 kV
Tube current (mAs)	50–150 mAs
Slice thickness	0.8–3 mm
increment	0.4–3 mm
collimation	64 × 0.625
Dose-length product	221.9–479.4 mGy cm

CT Scans before and after surgery where analyzed according to these steps:

1. Importing pre, and postoperative DICOM to Slicer 4.4.0
2. Defining anatomical landmarks of the mandibular condyle
3. Conversion of all RAS (Right, Anterior, Superior) coordinates corresponding fiducial marks (F) and migration into a.csv data set.
4. Calculation of the differences with the open source statistical programming environment R 3.1.1(33).

### Statistical Analysis

Descriptive statistics were used to summarize the clinical characteristics of the study cohort. Assessed variables were the intercondylar distance (F1 to F1′; F3 to F3′), and the position of the condyles before and after surgery by means of an angle measured between the left (Co si.) and the right condyle (Co dext.). All data where imported into MS Excel 15.6 for Mac from the Slicer 3D 4.4.0 program for statistic calculation.

We postulated that there is no significant difference in the condyle position after surgery. The position and the anatomical shape of the condyle where analyzed before and after surgery in a three-dimensional manner as described earlier. Then changes between the condylar position and condylar shape after surgery where analyzed and compared statistically. First, we performed a Shapiro Wilk Test to test whether the difference of the condyle position before and after surgery was within a standard distribution. If this was the case, we performed a paired t-test. If this was not the case, we performed a Wilcoxon-Mann-Whitney-Test. Statistical significance was defined as a two-sided p-value < 0.05. Statistical analysis of data was performed using the open source statistical programming environment R 3.1.1^21^.

## Results

A total of 16 patients with malocclusion who underwent orthognathic surgery met the eligibility criteria for this study.

All 16 patients were Caucasian aged 17–35, mean age 26,10 ± 5.1 years. Eight of them were women (aged 24.8 ± 5.3 ranging from 17.0 to 34.0 years) and eight were men (aged 27.3 ± 4.7 ranging from 22.2 to 35.7 years). There was no difference in age between men and women (Welch two sample t-test: −0.985, df = 13.723, p-value = 0.342).

14 patients with complex dysgnathia underwent bimaxillary treatment. Two patients underwent osteotomy of the maxilla only. Mandibular retrognathism was diagnosed in 11 patients (6 female, 5 male), demanding surgical advancement of the mandible. Five patients (2 female, 3 male) were diagnosed with mandibular prognathism and underwent a mandibular setback operation. Three patients underwent an additional genioplasty.

Analysis revealed no significant differences of the inter-condyle angle between bimaxillary and LeFort I osteotomy patients: Bimaxillary osteotomy patients showed an inter-condyle angle change of 3.3° ± 7.7 whereas LeFort I osteotomy patients showed an inter-condyle angle change of 2.8° ± 3.6. LeFort I osteotomy in comparison to bimaxillary osteotomy and mandibular set-back in comparison to mandibular advancement did not account for significant differences of inter-condyle angle change (see Table 3).

**Table 3:** Analysis of Variance of inter-condyle angle change (=postoperative intercondyle angle - preoperative intercondyle angle). Le Fort only and Angle class III vs. II were tested as factors.

Response: Inter-condyle angle change	Df	Sum Sq	Mean Sq	F value	Pr(>F)
Le Fort	1	0.41	0.405	0.0072	0.9337
Angle Class III vs II	1	47.59	47.590	0.8453	0.3746
Residuals	13	731.91	56.301		

Df: Degrees of freedom, Sum Sq: Sum of Squares, Mean Sq: Mean Square, F value: F-Statistic, Pr(>F): p-value.

### Inter- and extra-condylar distance

**Figure 2:**
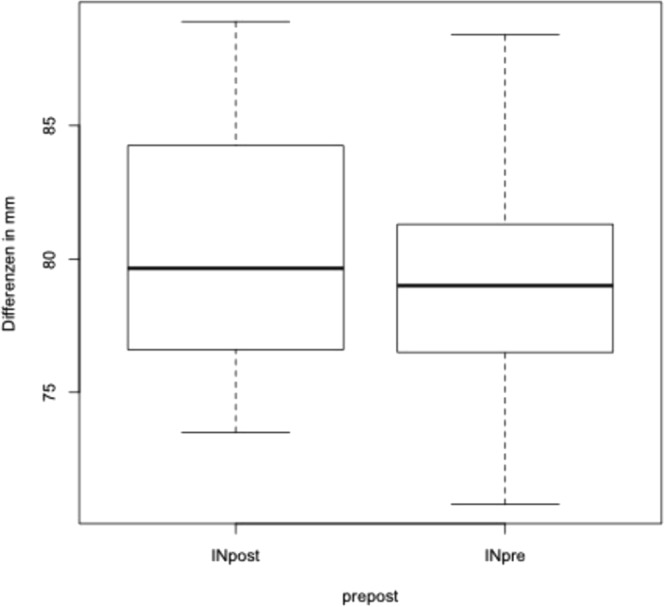
Analysis of changes of the intra condylar distance before and after surgery.

Distance between the most medial points of both condyles was measured before and after surgery to evaluate whether the inter-condylar distance was affected by the operation. The mean intercondylar distance was 79.0 mm before and 80.4 mm after surgery (see Fig. 2).

**Figure 3:**
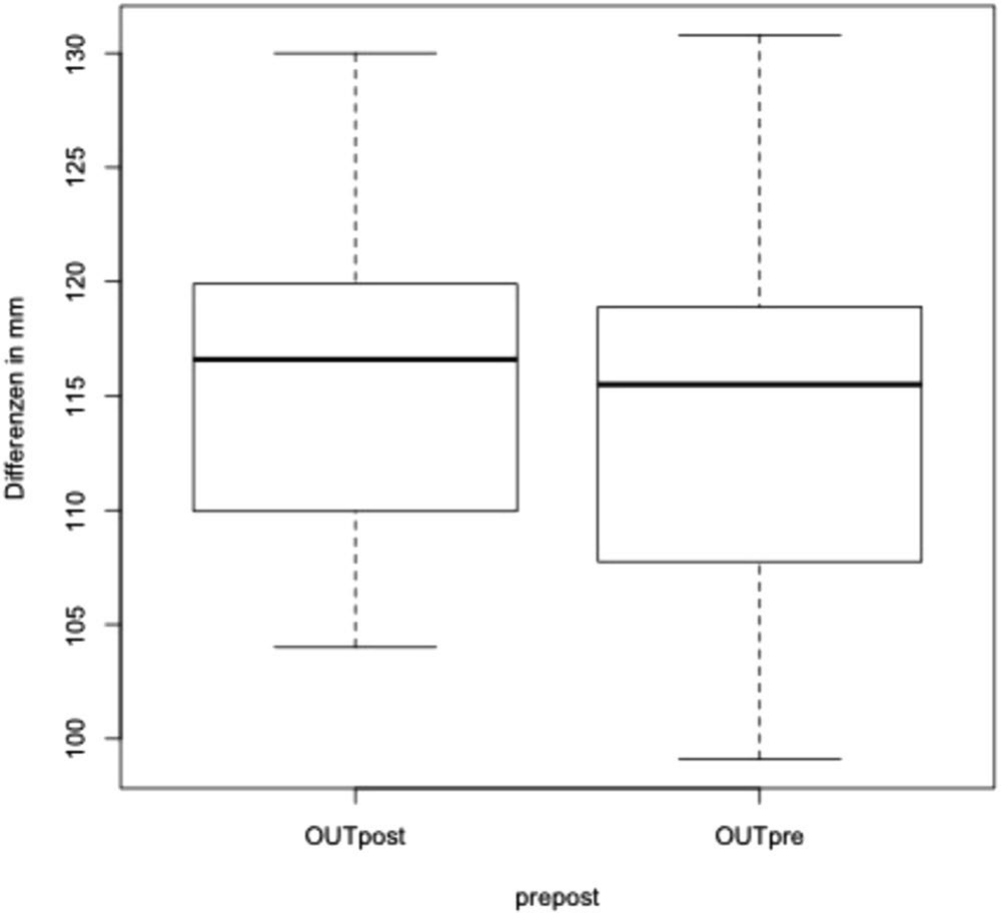
Analysis of changes of the extra condylar distance before and after surgery.

We performed the same calculation for the most lateral points of the condyles; the mean distance was 114.3 mm before, and 115.6 mm after surgery (see Fig. 3). The Shapiro Wilk test revealed that distances were not within a standard distribution (p = 0.0022). The paired T-test did not confirm significant changes between the distance of the lateral condyle position (p = 0.2114).

**Figure 4:**
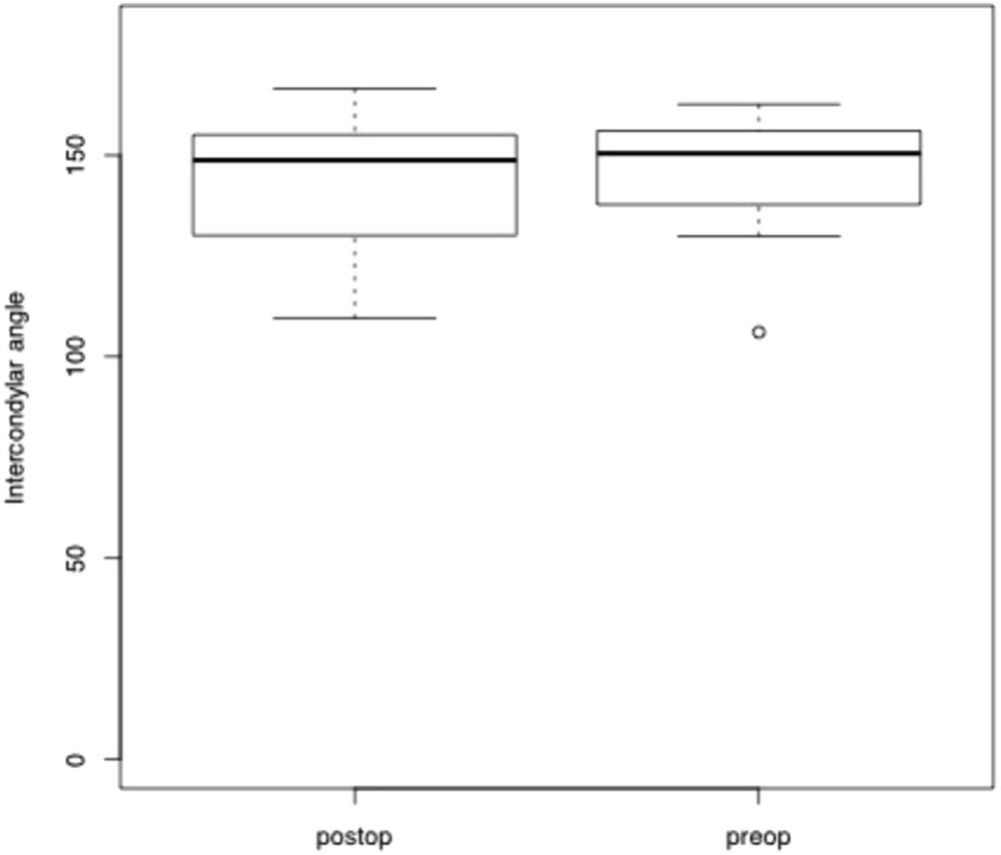
Analysis of changes of the intercondylar angle before and after surgery.

The angle between the condyles was measured at the crossing of the lines along the longitudinal axis of the condyle. The mean angle was 146.0 ± 14.8° before and 142.7 ± 16.0° after surgery (see Fig. 4). Since the inter-condylar angle did not follow a standard distribution (Shapiro-Wilk normality test: W = 0.90758, p-value = 0.0097). The angular change was compared pairwise before and after surgery, using a paired Wilcox signed rank test. The paired Wilcoxon signed rank test did not confirm significant changes between the angle of the lateral condyles before and after osteotomy (paired Wilcoxon signed rank test: V = 93, p = 0.2114).

## Discussion

The mechanisms underlying the correlation between pre- and postoperative condylar position changes have not been clarified yet. Hence, the aim of the present study was to examine the positional changes of the condyles in a group of 16 uniformly treated DFD patients with a follow-up of six months.

Numerous studies have shown that the amount of change in condyle position varies in each individual and is influenced by numerous factors, such as surgical procedure, experience of the surgeon and patient anatomy^3,10,14,15,22^. In the present study however, no significant changes in the position of the condyle were measured six months after surgery. Our findings are supported by Tabrizi *et al*., who observed that the distance of the condyle to the superior in the external auditory meatus in the coronal plane underwent significant movements compared to the preoperative position on both sides. Furthermore, they found that the condyle was displaced inferiorly in the sagittal plane one month after mandibular advancement with maxillary superior repositioning. Then, it moved superiorly approximately in the initial position. In the second measurement, a month after surgery, the condyle displaced laterally in the sagittal plane and repositioned to its original position after nine months. In the third measurement, the condyle displaced anteriorly one month after the surgery; then it positioned more posteriorly than its initial position nine months after the surgery. Changes in the sagittal plane were controlled by evaluation of condylar changes to the superior point of the external auditory meatus in the coronal plane^23^.

Our findings are also in line with the findings of a study by Chen *et al*., who found that the condyles tended to be positioned in a concentric position in relation to the glenoid fossa three months after the surgery and remained stable during the 1-year follow-up^24^. Contrarily, Harris *et al*. detected that 8 weeks after the SSO and mandibular advancement, most cases showed displacement of the condyle medially, posteriorly, superiorly, and angled medially^25^. Although these findings are contrary to the results of our study, they might be explained by the fact that their measurements were carried out 4 month earlier than ours.

Contrary to our results, some studies demonstrated inward rotation of the condyle after BSSO^26–28^. These contrary results might be explained due to different surgical techniques used during the operation, the use of different fixation systems after BSSO (rigid vs. semi-rigid vs. non rigid fixation), or different parameters measured at different times after surgery. Since our measurements where performed 6 month after surgery, we might not have detected positional changes of the condyle as physiologic adaptive bone remodeling, induced by the recovery of masticatory functions might already have taken place^29^.

Experimental and clinical studies have demonstrated that there is a close relation between condylar position in patients suffering from DFD and TMJ disorders^1–4,30–34^. Although OGS is a standardized and safe procedure, it has been shown that there is a correlation between OGS and TMJ symptoms in 2% of patients after surgery, especially in those patients who were already affected by TMJ disorders prior to surgery^7,34^. To prevent changes in TMJ position during surgery, several mechanical and computational devices have been developed to prevent the condyles from moving away from their original position, trying to address the correlation between OGS and TMJ symptoms^35,36^.

In this study no condylar positioning device was used, but surgery was carried out by two experienced surgeons to prevent extended condylar movement during the procedure and preserve the anatomical relation to reduce the influence on condyle anatomy and TMJ Function^37,38^. Our 3D analysis of condylar positional and volumetric changes shows, that there is no significant change in condylar position or anatomy after surgery. This finding is supported by current literature, which suggests that patients do not benefit from the use of condylar positioning devices^9,10,14,15,39^. In accordance with recent research we can postulate that there is only little change in mandibular structure and function after OGS^32,33^. This is supported by other studies, revealing that small positional changes of the condyle are not associated with early skeletal relapse^11^.

A unique aspect of surgery before orthodontic treatment is the possibility to evaluate the preoperative position of the condyle and the TMJ in its natural relation before orthodontic action. Although all measurements where done in a three-dimensional manner, this study is still subject to inherent limitations. All CT scans are prone to evaluation errors of condylar changes after orthognathic surgery. These errors are related to slice thickness, window level and width, matrix size, and rendering technique^40^. Another limitation is the small sample size, and the surgery first treatment concept as orthodontic treatment after surgery might have an impact on the position of the condyle. Another limitation of our study is that this method of measurement showed changes of intercondylar angles of 2.8 ± 3.6° in the LeFort I - only patients, although they underwent no relevant changes in the mandible. Consequently, this has to be interpreted as a measurement error or the effect of orthodontic treatment.

## Conclusion

The present study revealed the clinical significance of changes to the TMJ after OGS in patients with DFD. We were able to demonstrate that free hand condyle positioning during orthognathic surgery has little effect on the natural condyle position in patients without orthodontic treatment. Further studies are needed to confirm the findings of this study.

### Statement of Clinical Relevance

Freehand positioning of the condylar segments during a bilateral sagittal split osteotomy of the mandible in surgery first treatment concept, does not significantly change the position of the condyles.
